# Social Media Intervention Design: Applying an Affordances Framework

**DOI:** 10.2196/11014

**Published:** 2019-03-26

**Authors:** Megan A Moreno, Jonathan D'Angelo

**Affiliations:** 1 Department of Pediatrics School of Medicine and Public Health University of Wisconsin Madison, WI United States

**Keywords:** social media, health, adolescent, research

## Abstract

Social media interventions are a growing area of internet research, particularly for adolescent health. Researchers developing social media intervention approaches face the task of selecting a social media platform for their intervention. In this paper, we present the theoretical framework of affordances to help guide social media platform selection for intervention research. Affordances are a concept often used in fields associated with design and by those systematically studying the impact of a design of an object. Thus, the affordances approach is often used by those considering the impact of information technology and the design of social media platforms. Affordances are often described as properties of artifacts that can be recognized by users and contribute to their function or items that present an action possibility. We describe common affordances that can be applied to intervention design as well as current evidence and an intervention case example for each affordance. A scientific approach for the selection of the appropriate social media platform for a given intervention is an important research priority to advance the field of internet research.

## Introduction

### Background

Social media interventions are a growing area of internet research. This trend is illustrated by the growing number of systematic reviews examining social media interventions across different health topics including chronic illness and behavioral risk [1-3]. Social media as a platform for interventions targeting adolescents has technological benefits including ease of scaling up an intervention to reach large numbers of participants. Furthermore, social media has developmental salience for the adolescent population. Over 90% of adolescents report going on the Web every day, and almost a quarter of teens report that they go on the Web “almost constantly [4].” Adolescents have been dubbed *digital natives* given that they have grown up with access to computers and the internet from an early age [5]. Adolescents typically maintain a “social media portfolio” by using a number of different platforms including Facebook, Instagram, and Tumblr [4].

Researchers interested in social media intervention approaches are faced with many tasks in the design of an intervention including theoretical or clinical grounding, selecting an appropriate target population, and defining appropriate outcome measures. For a social media intervention, an important task is selecting a social media platform for the intervention. If investigators rely on familiarity with or popularity of a platform, these selection criteria lack scientific grounding and may introduce bias in study design. In this paper, we present a theoretical framework to help guide social media platform selection for intervention research. The goal of this framework is to increase the likelihood that the success of interventions hinges on replicable technological qualities as opposed to personal preferences or other spurious considerations. This paper is aligned with recent calls for more availability of information on processes and mechanisms to understand what makes Web intervention approaches feasible and effective [6]. We will first define our approach using affordances and describe a methodological approach for incorporating 4 common affordances into intervention design. Within each affordance section, we will include relevant evidence and a hypothetical case scenario. An evidence-based approach for the selection of the appropriate social media platform for a given intervention is thus an important research priority to advance the field of internet research.

### Affordances Defined

Affordances are a concept often used in fields associated with design and by those systematically studying the impact of a design of an object. Thus, the affordances approach is often used by those considering the impact of information technology and the design of social media platforms. Affordances are often described as properties of artifacts that can be recognized by users and contribute to their function [7] or items that present an action possibility [8]. Perceived affordances can also be conceptualized as “design aspects of objects that suggest to the user how the object should be used [7].” These definitions can feel somewhat opaque; however, a simple example of an affordance is that the design elements of a chair suggest to a person that the object could be used for sitting. A chair has 4 legs, a flat platform, and a straight back; these design affordances support the idea of a chair being an object designed for sitting. Importantly, although an individual’s goal or desire may change, an affordance does not evolve [9].

Given the rapid evolution of computer-mediated environments, many fields that study outcomes associated with this context have pushed researchers to utilize this framework for the sake of developing lasting and generalizable theory. Several scientific fields and disciplines have moved to discuss or encouraged the move to discuss technology not in terms of specific communication platforms but rather in terms of the affordances. These fields include management science [10], communication theory [11], education research [12], design research [13], information technology [14], organizational research [15], and tourism and marketing research [16]. The initiative is also present in clinical research as previous work has incorporated affordances related to utilizing social media to manage chronic disease [17]. Just as observational research benefits from enhanced clarity and meaning from the affordance framework [18], it is likely that intervention research will benefit as well.

### Benefits to an Affordance Approach in Social Media Intervention Research

There are several benefits to the application of the affordance approach for intervention design. First, an affordance approach provides a scientific framework for the selection of a social media platform. With this approach, a researcher can identify the necessary functionalities of an intervention such as key functions the platform should have or critical constructs linked to the behavioral or health-related theory that inform the intervention. These functions and constructs can then be used to match to the affordances of a social media platform. Second, this approach goes beyond the selection of interventions on the basis of the popularity of platforms. Given that the popularity of platforms can change, a public perception that social media is always changing is a concern that scientists may encounter. With an affordance approach, a scientist can describe the affordances needed for intervention and thus present potential platforms that would meet these criteria rather than relying on a single platform. If a successful intervention sees decline because a platform is losing popularity or becoming obsolete, an intervention designed around affordances can identify another platform that fits (or even design one). Finally, an affordance approach takes a step beyond the brand name approach of selection of social media platforms and changes the dialogue to be more closely linked to theory and technical function.

## Affordances to Consider in Intervention Design

Among the many affordances described in the literature, we will focus on 5 affordances that apply to social media. In what follows, we will describe the affordance category, provide evidence of how that particular technological affordance has been linked to psychological or behavioral change, and illustrate an intervention design targeted at an adolescent population.

### Identity Affordances

#### Definition

The first category of affordances of social media is identity affordances. Identity affordances include opportunities on social media platforms for *identity development and portrayal*. An example of a high-identity affordance platform is Facebook, on which users can upload a profile picture to identify themselves, list their “likes,” and share “life events.” Identity is further emphasized by the expectation on Facebook to use one’s real name (or a version of it). In contrast, Reddit is a low-identity social media platform on which users are typically identified by a chosen username. Furthermore, the emphasis on Reddit is more on what content a user contributes to group conversations rather than the personal identity of who posts.

It has been proposed that social media platforms that require fewer identity clues can allow users to take on new identities within different Web-based conversations, sometimes called *generative role taking* [19]. For example, an overweight teen working to become more physically active could create a new Twitter account called @TeenWhoLovesToRun dedicated to that identity. It is important to note that an extreme of this generative role-taking is represented by social media platforms on which users are anonymous, such as YikYak (shut down in 2017), and on which users could post with complete anonymity. A risk of fewer identity clues can lead to users taking on roles that are more dangerous, such as being aggressive, trolling, or bullying others.

Platforms that allow users to develop their identity may present benefits to adolescents in being able to explore and experiment with their identities, a critical developmental task of adolescence. However, by enhancing one’s own identity presence on a site, there are also risks of being identified by strangers. Thus, identity should be balanced with whether the site allows users to establish *privacy* settings. This attention to privacy may be more salient for interventions that involve illegal or stigmatizing behaviors such as substance use or mental health conditions. A balance of identity development and privacy protection is essential.

#### Evidence

The effectiveness of utilizing social media to help individuals shift identity or self-concept has been well established in experimental and observational settings. For example, when individuals were asked to present themselves as more extraverted on the Web, they, in turn, perceived themselves as more extraverted [20]. Furthermore, individuals asked to present themselves on the Web as loyal to a particular brand positively increased attitude toward that brand [21]. Finally, couples who post more relationship-related material on Facebook (thus cultivating a certain relationship identity on the Web) were more likely to still be together after 6 months [22]. Hence, there is empirical evidence to suggest that designing interventions on the basis of the ability of a platform to allow users to portray different aspects of identity can lead to positive change.

#### A Hypothetical Intervention

A social media intervention was being designed to promote physical activity among overweight adolescents. The intervention was intended to deliver positive messages to adolescents. The messages were designed to promote reflection on participants’ own skills and strengths to shift their view of themselves as active and fit, as well as providing prompts to engage in physical activity. The primary behavioral theory informing the intervention, which served as the root of the messages, was Self-Determination Theory [23]. In addition, findings of identity shift [20,24], the notion that individuals can internalize qualities that they present on the Web, supported this line of intervention. Thus, this intervention relied on the use of a social media platform that allowed identity affordances.

The researchers determined that low-identity affordance platforms such as Reddit and Twitter may not be the best fit for this intervention and considered the higher-identity affordance platforms of Instagram or Facebook. The intervention was then designed to take place on Facebook and include Facebook Badges and content that teens could incorporate into their own profiles. The rationale for the Facebook Badges approach was to allow teens to take salient intervention messages and integrate them into their own digital identities on Facebook.

### Social Affordances

#### Definition

Given the interactive nature of social media, it is no surprise that most social media platforms offer many social affordances. Social affordances include a sense of *belongin* g to a group such as a group focused on a particular interest, experience, social group, or religion [25]. Some social media platforms provide specific tools that allow the user to identify members of their group and enhance a feeling of belonging, such as tagging [26]. Hashtags (ie, content labeled with a # sign) are commonly used on Twitter and Instagram and can enhance belonging. When content is labeled with a hashtag, it is connected to all other content on that site that has also been labeled in that way. Thus, a hashtag can allow the creation of a community of users across different social networks who are connected by use of a particular topic or term. Any user on a platform can search by a hashtag and be connected to all content by others using that hashtag; thus, a community of those using that particular word or phrase can be connected. A positive example of belonging is teen cancer survivors who use a particular hashtag to connect to other teen survivors nationally to provide support and share similar experiences. Furthermore, social media may promote *network-informed associations* such as when Facebook suggests friends for a user on the basis of the user’s friends’ friends. This allows users to see how friends are connected to other people and see their interests [19].

An important task for adolescents to learn within the social media setting is how much information to share. A previous study found that intimate disclosures shared publicly can be judged as inappropriate [27]. Users must thus engage in *audience management* by monitoring and checking what audience is receiving their messages. This can impact how much personal information a teen is willing to share within a group-based intervention such as one using private Facebook groups. At the same time, users must also come to realize that their audience may not be exactly who they imagine it to be [28]. Assuming an audience different than one actually finds on social media may have unique psychological or social consequences. Finally, social media allows teens to experience and participate in *metavoicing* [19]. This term refers to how a social media user who posts anything in social media is engaging in a larger context including other people’s presence, profiles, content, and activities. Thus, an adolescent posting on Twitter is not merely voicing a single opinion but contributing to content that is already in that space and connected to others.

#### Evidence

The use of social affordances in health interventions specifically designed around this notion has already been shown to be effective in several areas. In a previous intervention, patients with non–small cell lung cancer who partook in an online support group, as opposed to those who only had access to internet articles, experienced fewer symptoms of distress [29]. Another intervention found that newly diagnosed breast cancer patients who received support from breast cancer survivors in an online forum reported improved quality of life and decreased depression [30]. Study trials are being designed to create Web-based portals for older adults who often suffer from isolation; early findings have demonstrated potential benefits [31]. Although not all researchers can design their own Web-based portal, this affordance can be utilized as it exists on already popular social media websites.

#### A Hypothetical Intervention

A social media intervention was designed to promote social support among teens with depression. The intervention was designed to promote peer-to-peer support among teens with depression, as peer support has received strong evidence as an effective intervention [32]. Thus, social affordances that promote peer-to-peer communication were a priority. Furthermore, pilot testing with teens led to the feedback that the social communication needed to be in private settings. The rationale for the platform selected was to utilize private groups in a platform that teens already visited.

Given that the teens already utilized Reddit, researchers decided to implement this intervention as a private group on Reddit. In addition to already being utilized by the participants, the platform had added affordances. There was no identity requirement for this platform, enabling participants to comfortably disclose more of their thoughts and feelings. The functional affordances of this message board allowed for the construction of messages at a time and rate that were comfortable to the participants.

In carefully analyzing the platform according to the affordance framework, the researchers realized that Reddit offers 1 emotional affordance that may be less beneficial: the ability to upvote and downvote messages. However, moderators of a given message board, the position that the intervention leaders will act in, have the ability to remove the ability of board members to downvote messages. By taking this action, the intervention platform was adjusted so that posts could only receive positive feedback.

### Cognitive Affordances

#### Definition

Cognitive affordances include using social media tools *to expand one’s learning*. Examples may include increasing awareness of global news events using Twitter or learning a new strength training exercise on YouTube. These affordances may be particularly useful for adolescents who may not traditionally get exposed to this information offline [7]. Cognitive affordances may also include the opportunity for enhanced creativity, such as on platforms such as Tumblr that allow customizable content.

Social media can also allow *reallocation of cognitive resources* by allowing a user to focus on 1 aspect of a person’s presentation at a time versus the many cognitive cues that emerge during a face-to-face conversation with a person. Face-to-face communication requires an adolescent to be cognizant of the complex interplay of words, tone, facial expression, and body language. In contrast, social media tools allow more simplified communication venues, such as Facebook messaging, which is text-only against a background of personal information. This type of communication requires fewer cognitive inputs and may be easier for some teens, but it can also lead to the potential for misinterpretation of information. For example, interventions that include humor or sarcasm in messaging may be at risk for misinterpretation without voice or visual cues. As a developmental task of adolescence is learning how to develop skills in communication, these tools may both help and hinder an adolescent’s journey.

Another cognitive affordance of many social media platforms is *triggered attending*. Triggered attending involves rejoining a Web-based conversation or responding to content when an automated alert informs the user to do so [19]. Examples include setting mobile phone alerts to trigger when specific users or friends post content. This triggered attending may be helpful to reengage adolescents at different stages of an intervention, but it could also be disruptive to adolescents who are engaged in other activities such as homework or driving.

#### Evidence

Similar to utilizing the social aspect of the internet and social media, the cognitive affordances of social media have begun to show promise. At the most fundamental level, social media has been shown to support informal learning at home [33], as well as be a source for those who seek health information on the Web [34,35]. More dynamically, aspects such as the reallocation of cognitive resources have been shown to allow users to reduce social anxiety associated with interpersonal interaction [36,37]. This reallocation can allow users to potentially present their best possible self, leading to more reciprocal self-disclosure and more intimate Web-based relationships [38]. Triggered attending was well documented in cell phone–based interventions, where the evidence can be simply expressed in the form of text message (short message service, SMS) reminders regarding patient appointments [39]. Given that mobile phones now produce notifications for social media in just as noticeable a fashion as SMS text messages, social media interventions are likely to benefit from such an affordance.

#### A Hypothetical Intervention

A social media intervention was designed to promote teaching of diabetes management skills among newly diagnosed adolescents. A rich media platform for teaching and a high cognitive affordances approach was a priority for the research team. The team determined that video was the best medium to teach basic skills, as evidence suggests that video is more efficient than text-based e-learning for practical or procedural skills sets [40]. Researchers worked with teens to create a private YouTube channel and new YouTube accounts for participants in the study as identity affordances were not critical to the study design. The research team was able to share videos to teach diabetes management skills, and participants were able to ask questions within the YouTube channel. Participants were also invited to exercise creativity and create their own teaching content to share on the private channel.

Even if participants were not inclined to create their own content, the functional affordances of this domain offered other routes to participation. Specifically, YouTube allows for the cultivation of video lists on profiles. Hence, participants were encouraged to cultivate a list of nutrition-related videos on each profile. This enabled 2 positive outcomes. First, participants and moderators were able to discuss the merits (or flaws) of each video to enhance the media and nutritional literacy of participants. Second, libraries of helpful videos were created for the participants to reference over time.

### Emotional Affordances

#### Definition

Emotional affordances include attributes of social media that can trigger or stimulate users’ emotional reactions [7]. For example, many platforms allow users to express emotion via the capacity to like or dislike content such as the “favorite” function on Twitter or “upvoting” or “downvoting” on Reddit.

Emotional affordances can also include *generating empathy* by seeing personal photographs or names alongside messages or news stories. It is common to see fund-raising efforts on social media that capitalize on emotional affordances by providing photos and personal stories. Research groups may seek to engage participants by sharing personal information about the researchers or providing photos of the research team. In contrast, another affordance of social media is *comparison.* Previous studies have illustrated that by examining other people’s social media profiles, users are at risk of feeling inadequate or envious [41].

#### Evidence

Relatively simple emotional affordances can prove to have distinct emotional outcomes. For instance, receiving Facebook likes on a post can make users feel socially supported [42], and photographs can affect judgments of personality more so than text in social media [43]. Moreover, experiments suggest that individuals already utilize social media to manage their emotions: when individuals were induced into a negative mood, they viewed social media profiles of individuals they perceived as less physically attractive or successful (downward social comparison) [44]. The effects of social comparison have also been evidenced in a lab-based health intervention experiment: individuals who received an appearance-based sun-protection intervention that included downward social comparison (ie, viewed photographs of individuals who had already experienced sun damage) were associated with less sun-protective behaviors compared with viewing the control condition. However, upward social comparison leads to a slight increase in sun-protective behaviors [45].

#### A Hypothetical Intervention

An intervention was designed for smoking cessation for older adolescents. The investigators wanted to share stories of older adolescents who had successfully quit to utilize emotional affordances to motivate and connect to participants. The investigators built a blog using a blogging website and promoted content via Instagram. A critical component of how content was shared with participants on Instagram was to use personal photos of each new blogger.

This intervention was grounded in notions of homophile, as people are more likely to adopt health advice offered by similar others [46]. Photographs were crucial for generating the emotional connection as photographs can have a greater impact on the judgment of a target than textual information [43]. Hence, it was uniquely catching for participants to see a picture of someone just like them who had successfully quit smoking.

### Functional Affordances

#### Definition

Functional affordances are principal functions that affect how social media messages are transmitted or saved. Although many of these affordances describe most social media platforms, an understanding of these affordances may be helpful for researchers to frame the key functional design elements of the tools needed for intervention.

Most social media platforms allow for *replicability* of messages, meaning that content can be reused by others. An example is the ability to “retweet” content on Twitter. This function can allow interventions to deliver “booster” doses of intervention messages by resharing previous content. Social media also allows messages to be highly *scalable*; there are numerous examples in the popular media of social media posts going “viral” and reaching far beyond the initial intended audience. For researchers, this can be an important consideration for ensuring that control group participants are not exposed to messages specific to an intervention if these are present on public social media.

Social media is also *searchable*; users can find specific content or people using a variety of Web-based means such as hashtags. Social media also provides *permanence* to messages by placing them in Web-based conversations or on profiles. Even on platforms that are intended to be ephemeral, such as Snapchat, content is generally stored or could be captured and saved by others. Social media allows for *unlimited composition time* before posting a message, in contrast with the back and forth of a typical face-to-face conversation. However, not all users utilize this affordance with every post. Finally, social media has varying levels of the user’s ability to edit once something is posted. Some sites allow for removal or editing of content easily; Facebook allows for revising or deleting posts. However, the speed of Twitter can lead to challenges with removing or editing content once it has been retweeted by others. These functions may be important to particular intervention approaches and be incorporated as explicit tools that are relevant to a given intervention.

#### Evidence

The replicability of messages in social media in the form of repeats, shares, or videos going viral is a goal of many health interventions, but it is not always achieved. For example, Twitter has been shown to be an effective means of recruitment for health research when a message is successfully retweeted by others [47]. At the same time, there is often no control over replicability: health intervention designers cannot always get videos to go viral as they might intend [48]. Hence, many functional affordances need to be considered for potential outcomes, both intended and unintended, but they are not always the best focus for a mechanism of change.

#### A Hypothetical Intervention

An awareness-raising intervention message focused on sexually transmitted infection testing was developed by a group of researchers. They successfully pilot-tested the approach and found that it increased awareness of and willingness to seek testing in a sample of adolescents. They now want to bring the intervention into practice and launch a social media campaign using their messages. They leverage Twitter for the intervention, knowing that retweets are a less-effort-intensive way to engage participants, and ask for retweeting of the message to increase its reach. They measure the number of likes, retweets, and impressions for their message over a 7-day period.

## Discussion

### Summary

The ever-changing landscape of social media sites can lead to challenges for researchers to apply scientific scaffolding to justify the selection of platforms for interventions. We present a research priority of applying an affordance framework for mindful and evidence-based selection of social media platforms to match intervention requirements and approaches. As indicated in these examples, thoughtful articulation of intervention outcomes and assessment of platform affordances can lead to an evidence-based selection of an ideal intervention platform, considering the affordances of a platform can help identify potential strengths as well as undesirable outcomes in an intervention and offer solutions. For instance, in an example intervention above, we identified the potential negative outcomes of a downvote on Reddit and thus removed that option within that particular intervention. There also exists a second potential pathway to more effective interventions: the design of an intervention that requires a set of affordances not yet present in current technology. Rather than a limitation, this represents opportunity in the area of technology. Previous computer-mediated communication research has offered suggestions to website designers to increase the happiness of Web-based daters and increase the instructional efficiency of educational technology [49], and new app developers need to be cognizant of the affordances of the products they develop. Identifying a grouping of affordances that can be beneficial but does not yet exist represents an opportunity for new platform development.

### Limitations

The affordances that we present here represent early efforts to define and apply affordances to adolescent health interventions. Conceptualization of affordances is an ongoing, iterative process across many research disciplines. Even in fields long interested in conceptualizing affordances, there is still debate around the definition and boundaries of each affordance [11]. However, the fact that this debate continues is evidence of the utility of this framework. Even if not perfect, it allows for purposeful selection and engagement across a broad and ever-evolving technological environment. We have presented several example interventions and named a few specific platforms as examples, but this neither implies that we endorse those platforms nor implies that all social media platforms are included in this paper.

Furthermore, in focusing this paper on an affordance approach, we recognize that intervention development takes into account many other factors beyond this theoretical approach. Appropriate targeting of a given platform to the participant group is critical. If your target demographic does not use a particular platform, this platform is not likely the one for your intervention. Platforms may also need to be considered on the basis of what behavior is targeted in the intervention and whether that behavior is one that is appropriate to discuss on that platform. Considering the flexibility or malleability of the platform selected is important; platforms with shifting features could change over the course of a study and impact results. Researchers reading this paper will want to consider the other feasibility and acceptability factors that they typically would in any given intervention planning, alongside their theoretical approach.

### Conclusions

Despite these limitations, the affordances approach provides a theoretical framework for selecting intervention platforms on the basis of specific criteria and functions. We present a research priority of applying an affordance framework for mindful selection of social media platforms to match intervention requirements and approaches. As indicated in these examples, thoughtful articulation of intervention outcomes and assessment of platform affordances can yield a complementary partnership that leads to health outcomes.

## Abbreviations

SMS: short message service
